# Supplementary figures and images for: Verification of the role of exosomal microRNA in colorectal tumorigenesis using human colorectal cancer cell lines
Source: PLoS One. 2020 Nov 11;15(11):e0242057. doi: 10.1371/journal.pone.0242057 (PMC7657557; doi:10.1371/journal.pone.0242057)

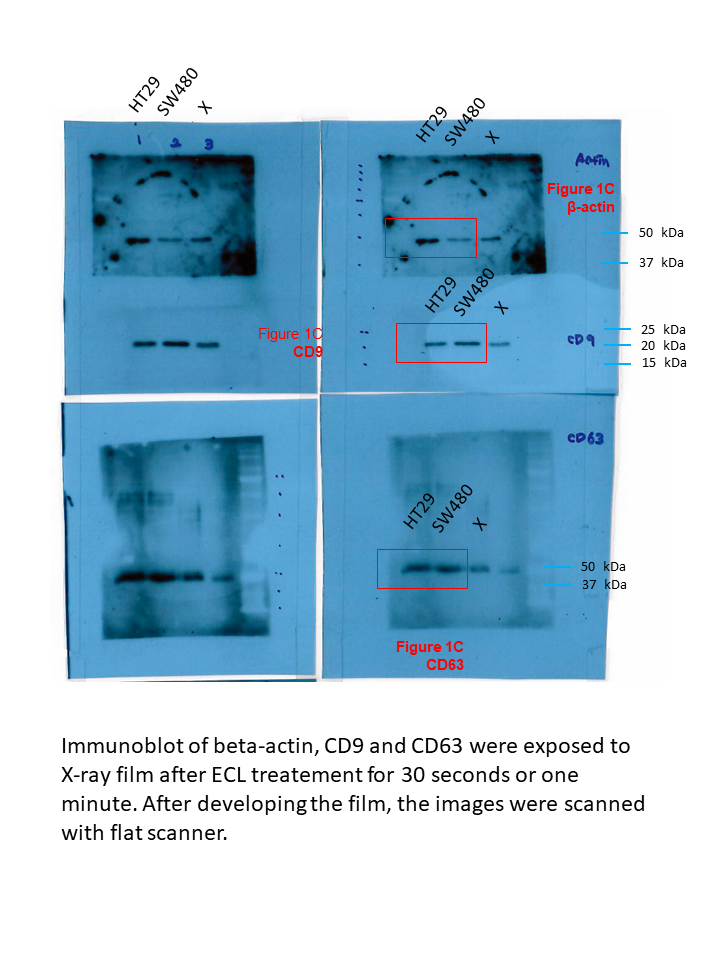

Supplement: S1 Raw images — (TIF) [file pone.0242057.s001.tif]
